# Benefits, challenges, and usability evaluation of DeloreanJS: a back-in-time debugger for JavaScript

**DOI:** 10.7717/peerj-cs.1238

**Published:** 2023-02-24

**Authors:** Paul Leger, Felipe Ruiz, Hiroaki Fukuda, Nicolás Cardozo

**Affiliations:** 1 Escuela de Ingeniería, Universidad Católica del Norte, Coquimbo, Elqui, Chile; 2 Shibaura Institute of Technology, Tokyo, Japan; 3 Universidad de Los Andes, Bógota, Colombia

**Keywords:** Web applications, Programming language, Software engineering, Usability evaluation, JavaScript, Debugger

## Abstract

JavaScript Web applications are a common product in industry. As with most applications, Web applications can acquire software flaws (known as bugs), whose symptoms are seen during the development stage and, even worse, in production. The use of debuggers is beneficial for detecting bugs. Unfortunately, most JavaScript debuggers (1) only support the “step into/through” feature in an execution program to detect a bug, and (2) do not allow developers to go back-in-time at the application execution to take actions to detect the bug accurately. For example, the second limitation does not allow developers to modify the value of a variable to fix a bug while the application is running or test if the same bug is triggered with other values of that variable. Using concepts such as continuations and static analysis, this article presents a usable debugger for JavaScript, named DeloreanJS, which enables developers to go back-in-time in different execution points and resume the execution of a Web application to improve the understanding of a bug, or even experiment with hypothetical scenarios around the bug. Using an online and available version, we illustrate the benefits of DeloreanJS through five examples of bugs in JavaScript. Although DeloreanJS is developed for JavaScript, a dynamic prototype-based object model with side effects (mutable variables), we discuss our proposal with the state-of-art/practice of debuggers in terms of features. For example, modern browsers like Mozilla Firefox include a debugger in their distribution that only support for the breakpoint feature. However DeloreanJS uses a graphical user interface that considers back-in-time features. The aim of this study is to evaluate and compare the usability of DeloreanJS and Mozilla Firefox’s debugger using the system usability scale approach. We requested 30 undergraduate students from two computer science programs to solve five tasks. Among the findings, we highlight two results. First, we found that 100% (15) of participants recommended DeloreanJS, and only 53% (eight) recommended Firefox’s debugger to complete the tasks. Second, whereas the average score for DeloreanJS is 71.6 (“Good”), the average score for Firefox’s debugger is 55.8 (“Acceptable”).

## Introduction

JavaScript is one of the most commonly used languages for developing Web applications. In fact, a survey conducted by Stack OverFlow (2022) showed that, for the past nine years, JavaScript has been the most used programming language. The popularity of Web applications has increased due to the number of standalone applications that have been migrated to the Web, to take full advantage of cloud and distributed services. Examples of migrated applications cover a wide range of domains, from PDF (Portable Document Format) to Microsoft Word documents conversion (Smallpdf, 2022) to Enterprise Resource Planning (ERP) Web applications (Oracle, 2022), and everything in between. We note that, as the complexity of new and migrated Web applications increases, they are more prone to the presence of flaws (*i.e., bugs*).

Detecting bugs represents one of the most time-consuming tasks in software development (National Institute of Standards and Technology, 2002), and the development of Web applications is no exception. To alleviate this task, alongside the proposal of programming languages and Integrated Development Environments (IDE), a large number of debuggers that provide many different features (Balzer, 1969).^1^ Even though multiple debugger alternatives exist, most practitioners still rely on classic breakpoint-based debuggers (Perscheid et al., 2017). Such debuggers allow developers to pause the execution of a program, allowing them to step through the program execution (*i.e.,* forward, into, or out of a given instruction). The advantage of this technique is to observe the values for selected program variables (Stallman, Pesch & Shebs, 2010; Dr. Racket, 2022). Other debuggers provide more advanced features like *navigation* through a program execution history (Pothier, Tanter & Piquer, 2007; Bousse et al., 2015; Barr et al., 2016), the *re-execution* of a program from a given execution point (UndoDB, 2022; Srinivasan et al., 2004; Bhansali et al., 2006; Choi & Srinivasan, 1998), or the *remote monitoring* of an execution (Session Stack, 2022; Raygun, 2022; TrackJS, 2022). However, the use of advanced 1Indeed, in 1966 a survey was published which showed features of debuggers for languages like Fortran, Lisp, and Algol (Evans & Darley, 1966).debuggers faces two problems. First, developers consider debuggers complex to use, opting to use *log* approaches with print-like statements (Beller et al., 2018). Second, most existing debuggers are *postmortem*. That is, the analysis of the program can only occur after the execution has taken place, and only for a single execution path (*i.e.,* a set of state values). As a result, classic debuggers only show the occurrence of a bug, forcing developers to execute their application multiple times to try to detect the bug over multiple value instances. This makes finding the cause of a bug difficult and time consuming, as the debuggers detect an instance of a problem, but provide no information about the underlying reasons for the program that occurred. We argue that offering the possibility of going *back-in-time* through the application’s execution, to replay a program using different values in an intuitive way, can improve understanding of the causes behind a bug.

**Figure 1:**
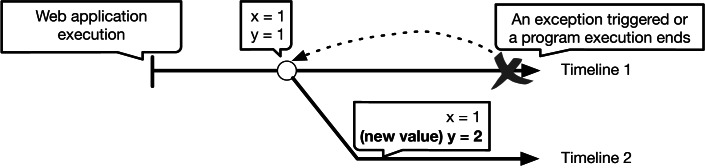
An overview of the DeloreanJS approach.

Inspired by the work on replay-based debugging (Tolmach & Appel, 1995) for Standard Meta Language (ML) (Milner, Tofte & Macqueen, 1997), this article presents a proof-of-concept usable (and practical) back-in-time debugger for JavaScript, named DeloreanJS. To realize back-in-time debugging, we introduce *timepoints*, defined as specific execution points of a Web application that developers can move to at any time. Timepoints are created explicitly by developers or implicitly whenever specified variables or object properties changed their values. Using timepoints, DeloreanJS enables back-in-time features that allow developers to: (1) navigate through an execution history by skipping through timepoints, (2) modify values associated with variables or object properties in a timepoint, and (3) resume the execution from a timepoint. Whenever a value is changed at a timepoint, the re-execution creates a new *timeline* (*i.e.,* a new execution trace for the application). The definition of timepoints and timelines is shown in Fig. 1. We present the details of timepoints and their implementation further in Section 3.

Additionally, we propose a specialized Graphical User Interface (GUI) for DeloreanJS to ease the usability of its features, in response to the complexity that back-in-time debuggers might introduce (*e.g.,* adding interactive timelines to navigate through the program’s execution history (Pothier, Tanter & Piquer, 2007)).

The development of DeloreanJS is based on the debugger for Standard ML, however, both debuggers are fundamentally different. On the one hand, Standard ML is a *purely* typed functional programming language, where there are no *side effects* (no mutable variables). However, JavaScript is a dynamically typed object-oriented programming language with higher-order functions and mutable states. To implement DeloreanJS, we use *continuations* (Friedman & Wand, 1984), to capture and store, as a first-class value, the current program control state of an application execution. Moreover, we extend continuations with *static analysis* (Cousot & Cousot, 1977) techniques to capture and store mutable objects (stored in the heap). This combination can completely capture the control and mutable state for JavaScript programs. Using the captured state, DeloreanJS creates timepoints that allow developers to go back-in-time to them and to resume execution from such points. If the stored state at the timepoints changes, the execution resumes in a new timeline with the changed state.

Using the back-in-time features, DeloreanJS allows developers to (1) improve understanding of a bug, and (2) experiment with hypothetical scenarios. DeloreanJS helps developers understand bugs as they can repeatedly modify variable values associated with a bug and resume an execution from the same timepoint, saving a large number of executions (*i.e.,* time). Additionally, developers can experiment with hypothetical scenarios of a Web application execution through the interactive user interface, allowing the exploration and evaluation of diverse timepoints with different variable values.

To validate DeloreanJS’ functionality (Section 4), we developed four different scenarios exhibiting the most important functionalities of DeloreanJS, which are to detect, understand, clarify, and experiment with bugs in JavaScript applications. The scenarios are taken from a Management Information System (MIS), and are made available in the online version of DeloreanJS (DeloreanJS, 2022).

Furthermore, to validate the usability of DeloreanJS, we conducted an empirical evaluation based on the System Usability Scale (SUS) approach (Brooke, 1996) (see the ‘Usability Evaluation’ section). Our evaluation consists of 15 computer science undergraduate students, with varying expertise levels, evaluating DeloreanJS in comparison to the built-in debugger in Mozilla Firefox (Mozilla Firefox, 2022). As a proof of DeloreanJS’ usability, all participants using DeloreanJS, recommended it; while 35% of the participants using Firefox’s debugger, recommended it.

In summary, our proposal constitutes an advancement in debugger research in three different dimensions, which we posit as our main contributions:

1. **Navigate through and resume from program execution points.** Unlike most debuggers for JavaScript, DeloreanJS allows developers to go back-in-time to specific execution points of a Web application and create new execution traces from a timepoint.
2. **Back-in-time debugger for a mutable object-oriented language.** DeloreanJS extends state-of-the-art features of back-in-time debuggers to enable the use of mutable states in the object-oriented programming paradigm.
3. **Usable user interface for a back-in-time debugger.** Advanced debugging features usually increase the complexity of using a debugger. DeloreanJS posits a user-friendly GUI to ease the navigation through timepoints and the generation of new execution timelines.

The rest of this article is organized as follows. The ‘Related Work’ section compares DeloreanJS to debuggers with existing approaches that have similar features. We then describe DeloreanJS and its crucial components in ‘Deloreanjs’. The ‘Validation: Deloreanjs in Action’ section presents our proposal in action through five examples. ‘Usability Evaluation’ presents the evaluation of DeloreanJS alongside a usability study based on the SUS approach. We end this article with a conclusion and describe the limitations of our proposal.

**Availability.** A proof-of-concept implementation of DeloreanJS with the tests presented in this article is available at http://pleger.cl/sites/deloreanjs (DeloreanJS, 2022). The source code is available from the following GitHub repository: http://github.com/fruizrob/delorean (revision 5f98bc6). Our proposal currently supports Google Chrome (Google Chrome, 2022) and Mozilla Firefox (Mozilla Firefox, 2022) browsers without any need for extensions or plugins.

## Related Work

JavaScript is a widely used language with active research (Vázquez et al., 2019; Leger, Tanter & Douence, 2013; Leger, Tanter & Fukuda, 2015; Zheng, Bao & Zhang, 2011; ten Veen, Harkes & Visser, 2018; Lui et al., 2018) and development communities (jQuery, 2022; Angular, 2022; McKenzie, 2022; RxJS, 2022). Currently, there are several debuggers proposed in the body of literature (Barton & Odvarko, 2010; JsBin, 2022; NodeJS Inspector, 2022), offering a wide set of features, such as modifying variable values while an application is running (*e.g.,* FireBug; Barton & Odvarko, 2010).

Table 1 shows a comparison of debugger features in approaches that consider the execution history of an application. This table shows 15 debuggers with their supported approaches and features. For each debugger, some features are supported (black circles), and some are not supported (white circles). In the last row, we compare these debuggers to our proposed debugger. Considering the approach of these debuggers, we can classify them to four groups:

**Table 1:** Feature comparison of DeloreanJS and related debuggers. The black circle is full support for the feature, a half black circle means a feature (or half of it) is emulated, and a white circle means that it is not supported.

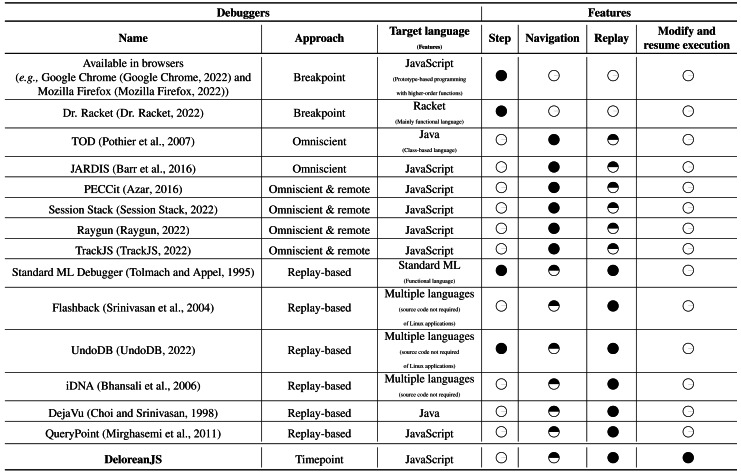

1. ***Breakpoint.*** Debuggers that use breakpoints are widely known. These debuggers allow developers to insert breakpoints to pause the program execution and start the debugging process when a program execution reaches a breakpoint. When the debugging process starts, a developer can step forward, statement by statement, or, as with the built-in Dr. Racket debugger, step back as well (Dr. Racket, 2022). Regarding JavaScript, modern browsers include a debugger in their distribution with breakpoint support (Google Chrome, 2022; Mozilla Firefox, 2022; Safari, 2022). More advanced JavaScript debuggers offer additional functionality. For example, FireBug (Barton & Odvarko, 2010), currently included in Mozilla Firefox, allows developers to modify a running Web application. Such features are desired in replay-based debuggers and are available in DeloreanJS. Although DeloreanJS does not directly support breakpoints, we may emulate this feature by inserting timepoints explicitly as shown in the following snippet: As the method implementation above shows, a breakpoint is added through the execution of the breakpoint method in the delorean object. In our proposal, the role of a breakpoint is to suspend a program’s execution to start the debugging process and work as a timepoint as well. Not surprisingly, the definition of this method is very direct because its implementation only inserts a timepoint and triggers an exception.
2. ***Omniscient.*** Omniscient debuggers, which have been implemented for languages like Java (Pothier, Tanter & Piquer, 2007) and xDSMLs (Bousse et al., 2015), record every event that occurs in the program’s execution, creating an execution trace history. In JavaScript, the JARDIS (Barr et al., 2016) debugger records the execution trace of a Web application and provides a GUI to navigate through this trace. Unlike DeloreanJS, omniscient debuggers are postmortem, meaning that it is not possible to go back-in-time to a point in the history of an application’s execution to resume the execution from a point with potentially modified variable values. Additionally, implicit timepoints in DeloreanJS allow us to emulate the history of the navigation through different variable values.
3. ***Omniscient and remote.*** JavaScript is commonly used to build Web applications^2^ 2Apart from the Web, JavaScript is currently used in other development environments, for example, on the server side with NodeJs (NodeJS, 2022) and in window managers of Linux-based operating systems (Gnome, 2022).that are running on devices with different hardware and software characteristics (*e.g.,* smartphones, tablets, notebooks). Given a device’s fragmentation, bugs may appear from different configurations and may not have been tested by developers due to scarse development environment. To overcome this difficulty, JavaScript developers use debuggers that remotely monitor the execution of applications (Session Stack, 2022; Raygun, 2022; TrackJS, 2022; Azar, 2016). Similar to omniscient debuggers, omnisciente and remote debuggers record and send the occurrence of each event to a developer over the network. Although these debuggers allow developers to analyze the execution traces of different users in real-time, they do not offer the possibility to navigate back-in-time through timepoints (*i.e.,* execution points) as is possible in DeloreanJS.
4. ***Replay-based.*** A deterministic replay tool (UndoDB, 2022; Srinivasan et al., 2004; Bhansali et al., 2006; Choi & Srinivasan, 1998) re-executes a program with the exact behavior of the original program execution. Some researchers have adapted this kind of tool to create replay-based (or reverse) debuggers. For example, Jockey (Saito, 2005) allows developers to replay program executions from specified “checking points,” to analyze program behavior from such execution points. Using continuations, the Standard ML (SML) debugger (Tolmach & Appel, 1995) provides a timepoint-like feature. However, SML is fundamentally different from JavaScript due to the support of mutable objects. QueryPoint (Mirghasemi, Barton & Petitpierre, 2011) is a JavaScript debugger with replay tools that allows developers to go back-in-time to the last change of a specific variable that could have an incorrect value. Apart from using timepoints to resume an execution with different variable and object property values, with DeloreanJS developers can use timepoints with watched variables to emulate a similar behavior to QueryPoint.

Runtime verification tools (Meredith, 2012) are not strictly defined as debuggers, nonetheless, these tools have a behavior similar to that of DeloreanJS given that errors can be detected at run time. Available runtime verification tools include PQL (Martin, Livshits & Lam, 2005), PTQL (Goldsmith, O’Callahan & Aiken, 2005), and JavaMOP (Meredith et al., 2011; Chen & Roşu, 2007). These tools allow developers to express the complex patterns of an application’s execution, for example, detecting access to an item that is not available in an array because another execution thread removed this item. Unfortunately, similarly to previous debuggers, these tools cannot resume an execution from a specific point of the computation history with different variable values.

## DeloreanJS

**Figure 2:**
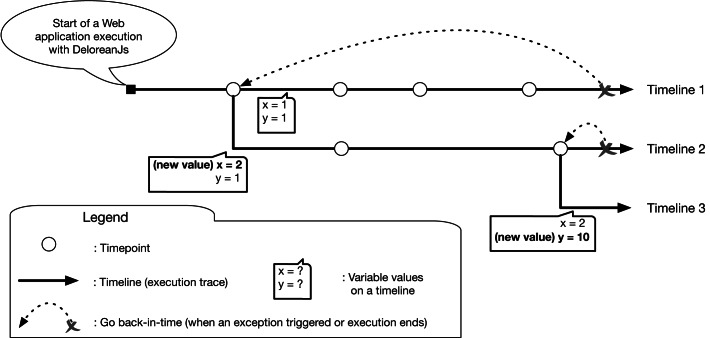
Multiple execution traces of an application with DeloreanJS.

**Figure 3:**
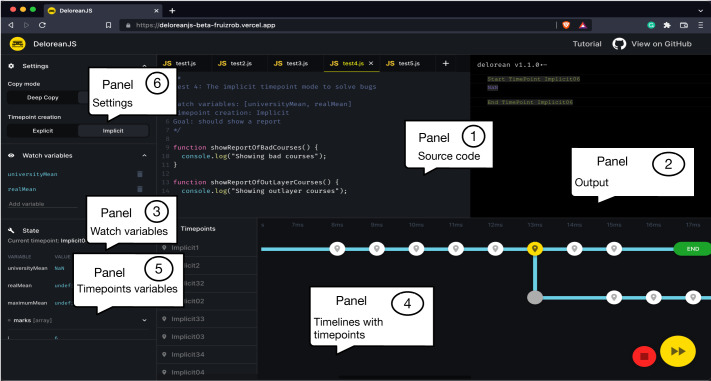
A screenshot of DeloreanJS as a Web application.

Unlike current JavaScript debuggers, DeloreanJS uses a *back-in-time* approach. Figure 2 shows the multiple execution traces, named *timelines*, that can be created from the timepoints inserted into a Web application. When a developer inserts a timepoint, it is possible to go back-in-time to that timepoint when the execution of an application is stoped due to an exception, or the regular execution flow ends. When we replay the execution from the timepoint, a new timeline is created. Each timeline represents a different execution trace, for example, the variables x and y in Fig. 2 contain different values. Figure 3 shows a screenshot from the current version of DeloreanJS (DeloreanJS, 2022). As shown in Fig. 3, the GUI is composed of six panels (inspired by Visual Studio Code; Visual Studio Code, 2022). Panel 1 allows a developer to write a JavaScript program to debug. Panel 2 shows the output of the execution. Panel 3 is used to add variables to *watch* by DeloreanJS. Panel 4 shows the *timepoints* with their corresponding *timelines*. A timeline, a specific execution trace, is created while the Web application executes; for example, Fig. 2 shows two timelines, where one is created from resuming the application execution in a selected timepoint. Panel 5 shows variables and objects that a timepoint captures. The values of these variables and objects can be modified by developers before resuming the execution. Panel 6 shows two configuration options to define the types of timepoints to use (cf. “Inserting and Using Timepoints” section).

 The Related Works section described existing back-in-time debuggers. However, the distinguishing features of JavaScript, like mutable and dynamic object-oriented programming with higher-order functions, introduce new challenges, not addressed by existing debuggers. For example, dealing with the mutable state of objects. The next section presents how we address these challenges in DeloreanJS.

### Creating timepoints

**Figure 4:**
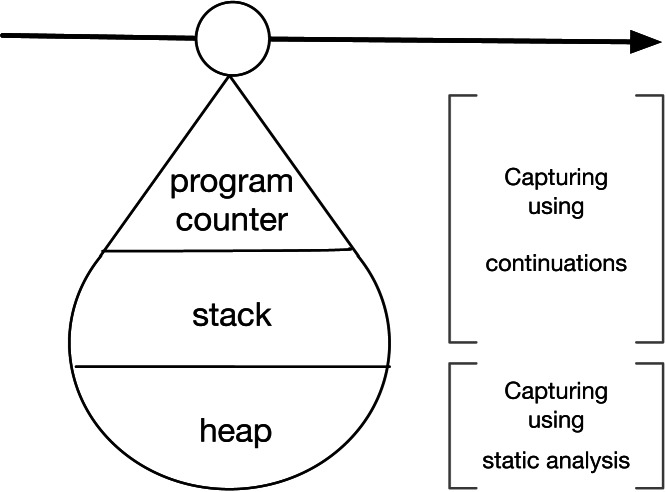
Composition of timepoints, created using continuations and static analysis.

To go back-in-time to a specific point in the execution of a Web application, the timepoint abstraction is crucial. This is because a timepoint captures and stores the control state of a Web application in terms of the: *program counter*, *stack*, and (partially) *heap*. Figure 4 shows how we capture the program counter and stack using *continuations* (Friedman & Wand, 1984). Variables from the stack and heap are captured using *static analysis* (Cousot & Cousot, 1977).

#### Capturing the program counter and stack

**Figure 5:**
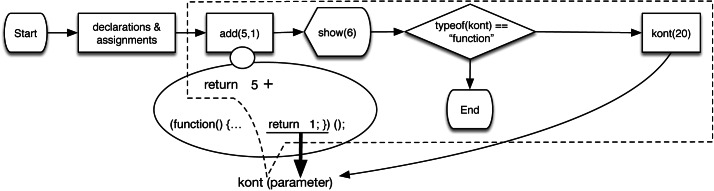
The process flow diagram of listing 1 that illustrates the use of continuations. The kont continuation uses a parameter to replace the return value of the anonymous function within add (figure taken from Leger & Fukuda (2017); Leger, Fukuda & Figueroa (2021); https://doi.org/10.1145/3019612.3019783).

To understand how to capture and store the program counter and stack, we offer a brief explanation of continuations (Koppel, Scherer & Solar-Lezama, 2018; Cong et al., 2019; Cong & Asai, 2016), which are pivotal to fulfilling this task. Functional programming languages, such as Scheme (Kesley & Rees, 1995), provide an abstraction named *continuation*. This abstraction captures the program counter and stack of a functional program and stores them as a *first-class value*, that is, a value that supports assignment and invocation operations (*e.g.,* functions in JavaScript). When a continuation is created, this can be invoked to replace the current execution of a program with the execution stored in the continuation. Unwinder (Long, 2022) is a third-party library in JavaScript that supports continuations through a function called callCC. Although this library is not being maintained, Unwinder provides the necessary functionalities to DeloreanJS. We exemplify continuations with Unwinder using a piece of code that captures the execution of a function that adds two numbers:

Figure 5 shows the workflow of Listing 1. Line 5 shows a continuation kont created before adding the y variable. This execution capture occurs on line 9 when the add function is called. The result of add is passed to show, and the number 6 (6 = (*x* = 5) + (*y* = 1)) is shown. Line 10 invokes the continuation stored in kont with the parameter 20, resulting in the return value of the anonymous function between lines 5–6 as 20 and not 1. As a result, 25 (25 = (*x* = 5) + (*y* = 20)) is displayed. Note that the *if expression* statement (line 6), and the *if statement* (line 10) are used to differentiate between the creation of a continuation and its invocation. A continuation is a function when it is created (line 5); and when the continuation is invoked, it is bound to the value passed as a parameter (*e.g.,* the value 20 in our example).

#### Capturing variables with their values

A timepoint needs to capture and store the variables found in a heap. However, continuations do not do this. To capture heap variables and values, DeloreanJS statically analyzes a Web application before running it. As JavaScript is single-threaded, the capture of heap variables does not need to deal with issues related to *data races*, as is the case in multi-threading languages like Java (Kimball & Grossman, 2007; Warth & Kay, 2008). For example, to manage situations where two threads modify the same shared variable, v1 = 5 and v2 = 2, and we use a timepoint, which would restore the variable value for only one thread.

**Figure 6:**
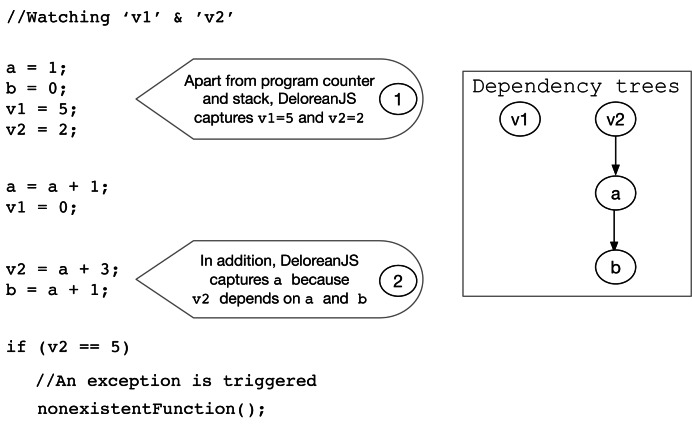
The two steps to capture and store watched variables (*e.g.,* v1 and v2) with their dependencies (*e.g.,* the a variable because it modifies v2).

The static analysis allows DeloreanJS to *instrument* the source code to store (during the execution) the values of *watched variables*. Figure 6 exemplifies the two steps in our static analysis. Considering a lexical scope strategy, Step 1 captures and stores the values of defined watched variables (*e.g.,* v1 and v2). In Step 2, the static analysis also captures and stores the variables that modify watched variables, *i.e.,* that have dependencies to watch variables (*e.g.,* a and b). As a result, DeloreanJS creates a *dependency tree* for each watched variable, where a node contains a variable that (transitively) modifies the watched variable (*e.g.,* the two trees shown in Fig. 6). This last step follows a *reactive programming* (Wan & Hudak, 2000) strategy and is necessary to ensure watched variables evolve consistently with the variable values when an application resumes from a timepoint. For example, in Fig. 6, if the a variable is not captured, then the v2 variable would have a different value when the application resumes its execution from a timepoint. To implement these two steps, DeloreanJS first creates an Abstract Syntax Tree (AST) from the source code. Using the AST, DeloreanJS applies the *Visitor* (Gamma et al., 1994) design pattern to visit every node of the AST to: (1) create a dependency tree for each watched variable, and (2) capture and store it at runtime any modification to variables in the dependency tree.

If watched variables are associated with objects, developers can select between a *shallow* or *deep* copy of objects in DeloreanJS’ user interface. The first option only copies the references to other objects, while the second option clones these objects. Both options contain a tradeoff that is necessary to consider. On the one hand, if the shallow copy option is used, developers may not keep an exact version of an object through the time, *i.e.,* developers can resume an execution from a timepoint with inconsistent memory (*e.g.,* object properties with future values). On the other hand, if the deep copy option is used, memory usage significantly increases, and it is possible that references of nested objects may not be changed (*e.g.,* modification of an object reference in a place that our debugger is not supervising). In the current implementation, the DeloreanJS’ user interface allows developers to choose between the two approaches.

### Inserting and using timepoints

Developers can *explicitly* insert timepoints using the delorean.insertTimepoint(String) method, and DeloreanJS can *implicitly* insert timepoints when watched variables and their dependencies change their values. To insert implicit timepoints, the debugger instruments the source code before the execution, to add a timepoint every time that a variable of any dependency tree changes its value (Fig. 6).

**Figure 7:**
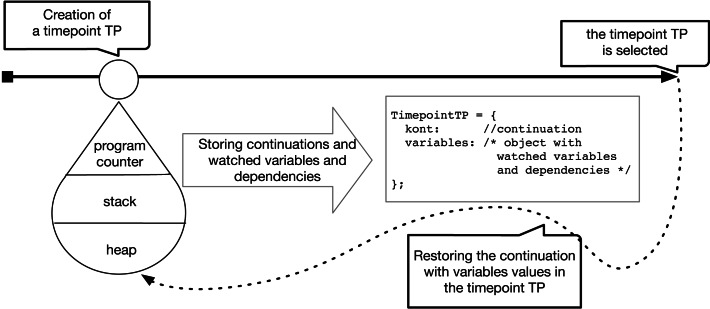
Insertion of a timepoint and its usage.

Figure 7 shows what happens when (1) a timepoint is inserted, and (2) the inserted timepoint is used. When a timepoint is inserted DeloreanJS creates and stores a Timepoint object, which contains a new continuation and an object that stores the watched variables with their dependencies. When a developer selects a specific timepoint (*e.g.,* TP) using DeloreanJS’ user interface, the debugger invokes the continuation stored in the timepoint and subsequently modifies the values of the watched variables along with their dependencies.

## Validation: DeloreanJS in action

This section introduces the main functionalities of DeloreanJS and its inner workings through five examples extracted from a MIS (Laudon & Laudon, 2016) that manages student grades at a university. To gradually introduce the debugger, the complexity of the examples is incrementally increased.

### Bug detection and fixing

A common task for a MIS is to calculate the final grade of a student, according to an evaluation strategy assigned to a course. We want to be able to identify errors, in a meaningful way, whenever this calculation cannot take place. Listing 2 shows an example of how to manage errors during the calculation. In this example, we calculate the final grade for a student of the course “Algebra.”. Here, an exception is triggered because the variable “courseName” contains an incorrect name for the course (“Al**gg**ebra”), which does not have an evaluation strategy assigned to it. Although the goal of DeloreanJS is to detect bugs, we can also use our tool to fix bugs at runtime. To do so, a developer must first add the courseName to the list of *watched variables* using the user interface; then the developer inserts an *explicit timepoint* (line 4) to be able to go back-in-time to it. When an exception is triggered, the program stops its execution. As we are attempting to invoke a function that is not in the evalStrategies array (line 9), the developer can select a timepoint such as StrategyNotFound using the DeloreanJS user interface and change the courseName value to the correct course name: “Algebra.”. Finally, the developer can resume execution from the selected timepoint for a successful calculation.

### Improve the understanding of a bug

Another desirable feature of the MIS is to generate a report that contains the average grade of all courses at a university. This feature is implemented in Listing 3. Similar to the code in Listing 2, the course “Algebra” is misspelled. However, this scenario is more complex as the exception is triggered within the execution of the loop, and has many possible iterations (*e.g.,* there can be more than 1,000 courses per semester). Knowing which iteration and why it triggers an exception in a loop can be an extremely time-consuming task for developers. The use of *breakpoints*, available in existing debuggers, does not necessarily ease the task at hand as breakpoints do not react to an exception, but react to the execution of a statement. In the case of a loop, such statements may be executed several times, having to stop at each of them. For this example, DeloreanJS allows developers to save the execution state from many executions (*i.e.,* loop iterations), and reuse the execution context of the application (*i.e.,* go back to a specific iteration and try different values). This is helpful information for developers to understand the bug. In Listing 3, a DeloreanJS timepoint is inserted for each iteration of the loop while the program is executing. When an exception is triggered, a developer can go back in time to any iteration of this loop to find the causes of a bug, improving their understanding of the reason for the bug. Again, in this example, we observe that DeloreanJS allows developers to watch and modify objects and their properties (*e.g.,* courseNames) to explore execution alternatives.

### Clarify unexpected results

A MIS has to administer the personal information of students, such as their name or birth year. Student information can be used to generate reports or aggregate information like the students’ average year of birth. Listing 4 shows a piece of code that can be used to calculate students’ average year of birth. In JavaScript, when there is a variable without initialization, this variable is bound to undefined (*e.g.,* Guillermo’s birth year). Additionally, JavaScript does not trigger an exception when arithmetic expressions operate with undefined, the language just returns NaN (Not a Number). The average variable of this listing ends up with a NaN because of an undefined value in Guillermo’s birth year. As a bug is an unexpected behavior (not only an exception), developers using DeloreanJS can employ timepoints when a program execution ends. Listing 4 inserts a timepoint for each student in the students array. Developers can navigate through these timepoints to find the iteration step when average goes from a numeric value to NaN, *i.e.,* when i = 4.

### Experiment with hypothetical scenarios

Experimenting with several hypothetical scenarios without the need to re-run an application from the beginning may save time for testers. Developers can use DeloreanJS to experiment with different execution scenarios by reusing timepoints with different values for watched variables. We illustrate this feature through three potential reports that this MIS can show depending on the value of the realMean variable, as shown in Listing 5. Using DeloreanJS with explicit timepoints triggered by exceptions, a tester can (re)use the timepoint TestingDifferentResults to modify the value of realMean and explore the behavior of the system when it displays different reports.

### Revisiting: improve the understanding of a bug

This does not strongly highlight its benefits. DeloreanJS’ user interface allows developers to activate the *implicit timepoints* option, so that timepoints are automatically added each time that a watch variable is modified. With implicit timepoints, a developer can *navigate* in the computation history of a program through the selection of timepoints that represent value modifications of watch variables. Other proposals (Pothier & Tanter, 2009; Barr et al., 2016) have shown the benefits of navigating through a program’s execution history to understand a bug; this is mainly because it is possible to find when a variable is bound to an unexpected value. For example, Fig. 3 shows the execution history through the timepoints with their associated timestamps; in each timepoint, developers can watch variable and object property values at that execution point. We illustrate the use of implicit timepoints for navigation through the second example of this section, when we attempt to generate a report that contains the average grade of all courses at a university. Without adding any call to a DeloreanJS method, the developer is able to watch the evolution of variable values in each iteration of the (long) loop, and, of course, resume the execution from a selected timepoint.

**Figure 8:**
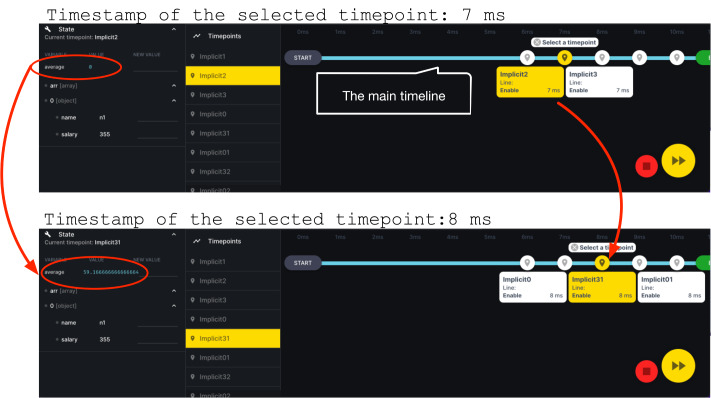
Two different execution points of an application execution, 7 and 8 ms; where the average variable changes its value.

Figure 8 illustrates the navigation that a developer can use with DeloreanJS. The figure shows the evolution of a variable named average in two different execution points, 7 and 8 ms (ms), that are contained in two timepoints. Whereas the value of average is 0 at 7 ms, this value changes to 59.1 at 8 ms. As many timepoints can be created at the exact millisecond, the user interface groups these timepoints in one point to simplify the interface.

### Summary

We have presented DeloreanJS through different concrete examples, which show the use of explicit and implicit timepoints to deal with bugs. Using timepoints, we have shown how to modify values of variable or object properties using a Web interface while an application is running. Although we use the first three examples with explicit timepoints, the usefulness of DeloreanJS comes from employing implicit timepoints, as developers can navigate through the evolution of values in the execution history of a Web application.

## Usability Evaluation

We evaluated and compared the usability of DeloreanJS and Mozilla Firefox’s built-in debugger (ex-Firebug (Barton & Odvarko, 2010)) using the SUS (Brooke, 1996) to detect bugs in five pieces of code written in JavaScript. The SUS approach has been widely used in different contexts (Bangor, Kortum & Miller, 2008; Derisma, 2020; Vlachogianni & Tselios, 2021) because of its quick and adjustable use (Brooke, 2013). In this section, we first describe the inspiration for DeloreanJS’ GUI, then we present the usability evaluation setup with its results.

### Graphical user interface

As mentioned in Section 4, DeloreanJS’ GUI is mainly inspired by Visual Studio Code (Visual Studio Code, 2022), which provides a familiar user interface for developers.^3^ 3The most used IDE in 2021 (Stack OverFlow, 2022).To interact with a timeline, we borrow the interface used in TOD (Pothier & Tanter, 2009) and other IDEs (Field et al., 2022). Additionally, as Fig. 3 shows, we include the timepoint interactions, the support of multiple timelines, and a panel to modify the property values contained by a timepoint. Readers can check out DeloreanJS’ GUI on its website (DeloreanJS, 2022).

### Evaluation setup

Table 2 shows the characteristics of the evaluation participants. We invited 30 undergraduate students from two computer engineering programs (from Universidad Católica del Norte—Chile, and Universidad de los Andes—Colombia). All participants are in their 4th or 5th year of the program and have 1–3 years of experience in JavaScript development. These participants analyzed five pieces of code (tasks), which are available in the DeloreanJS website (DeloreanJS, 2022). The task complexity is incremental, starting with the execution of the easiest to the most complex task (Table 3). The evaluation splits the participants in two groups, 15 students used DeloreanJS, and the remaining 15 used the built-in Mozilla Firefox debugger. After executing the five tasks, all participants filled out a Google Form survey that contained a set of questions. The evaluation was carried out in two online sessions–one for each university.

### Results

The results of the evaluation show that 100% of the participants using DeloreanJS (15) recommend it as an effective tool to debug JavaScript Web applications. 53% of the participants using Firefox’s debugger recommend it to debug Web applications. In this section, we present and compare different charts that illustrate he participants’ use of DeloreanJS. After presenting these charts, we briefly describe and apply the SUS approach to DeloreanJS and Firefox’s debugger. Participant responses used to create the charts and SUS evaluation are anonymized and available at http://pleger.cl/sites/deloreanjs/results.html (responses in Spanish and translated to English).

**Figure 9:**
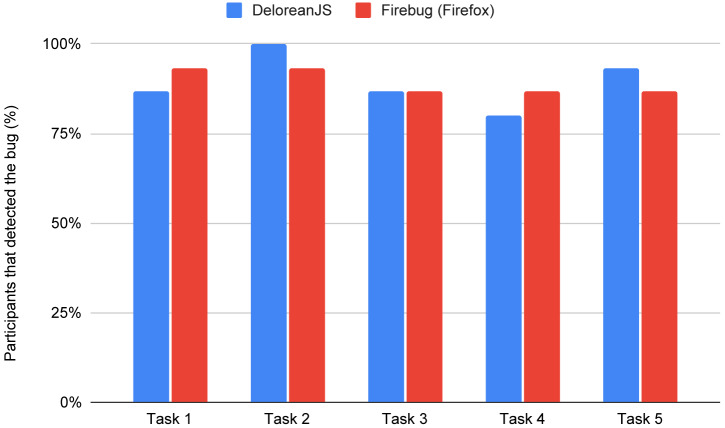
Percentage of participants that detected the bug per script for DeloreanJS and Firefox’s debugger.

***Percentage of participants that detected a bug.*** Figure 9 compares the percentage of participants that were able to detect a bug using each of the platforms debugger: DeloreanJS, and FireFox. For both debuggers, most participants (over 75%) could detect the bugs for all tasks. Note that all participants using DeloreanJS could detect the bug in task 2 (Listing 6) while only 93% of the participants could detect the bug using FireFox’s debugger. The difference in success rates may be because the piece of code in task two presents an unexpected behavior (NaN as a result) and not a runtime exception. Participants using DeloreanJS could use timepoints to find the moment when the result becomes NaN.

**Table 2:** Participants’ profiles per debugger.

**Debugger**	**Participants’ profile**	**Number**	**Universities**
Firefox	Undergraduate Students	15	University of the Andes (Colombia) - Universidad Católica del Norte (Chile)
DeloreanJS	Undergraduate Students	15	University of the Andes (Colombia) - Universidad Católica del Norte (Chile)

**Table 3:** Tasks for participants.

**Id**	**Description**
1	Detect a simple bug
2	Detect a bug into a loop
3	Experiment with different and hypothetical scenarios
4	Detect a bug using implicit timepoints
5	Detect a bug in advanced structures

**Figure 10:**
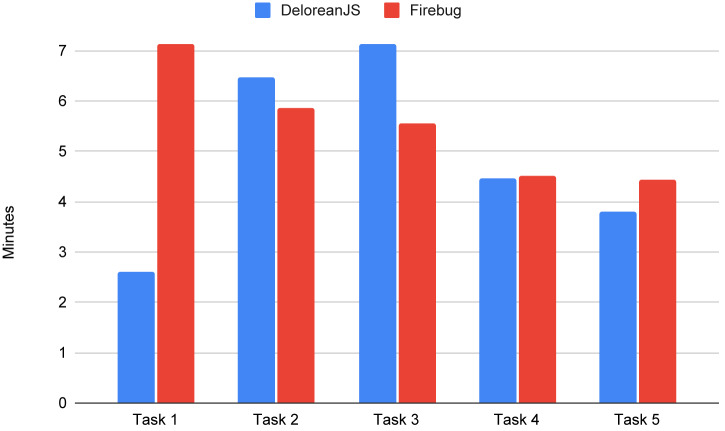
Average time (minutes) that took to the participants to detect each bug.

***Average time to detect a bug.*** Figure 10 shows the average time per task that participants used to detect the bug. In the first task, participants using DeloreanJS, detected the bug significantly faster than the participants using Firefox’s debugger, with a net difference of 4 min. In the remaining tasks, the average time to solve each task is similar for both debuggers. It is possible that the difference in time required to locate the bug in the first task is related to the fact that the first time participants used either debugger, and Firefox’s debugger seems more complex (cf. Section 5.3.1).

#### Usability

To evaluate and compare DeloreanJS with Firefox’s debugger in respect to their usability, we employed the SUS approach (Brooke, 1996). With the SUS approach, a set of participants that used a product, service, or application, are asked to score ten items using a Likert scale (Albaum, 1997) of five levels (from “Strongly agree” to “Strongly disagree”). The ten items are presented as statements that a participant scores:

• “I think that I would like to use this system frequently”
• “I found the system unnecessarily complex”
• “I thought the system was easy to use”
• “I think that I would need the support of a technical person to be able to use this system”
• “I found the various functions in this system were well integrated”
• “I thought there was too much inconsistency in this system”
• “I would imagine that most people would learn to use this system very quickly”
• “I found the system very cumbersome to use”
• “I felt very confident using the system”
• “I needed to learn a lot of things before I could get going with this system”

**Figure 11:**
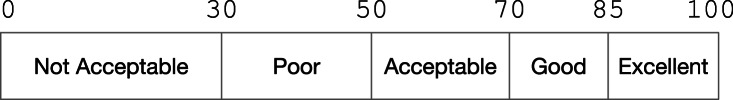
Range of values to determine how the usability is a user interface according tothe SUS approach.

To calculate a global score, we follow a three-step procedure. The global score is in the range of 0–100, which determines its usability according to Fig. 11.^4^ 4On the Web, few variations in the ranges can be found to classify the user interface.

1. Add up the total score for all odd-numbered questions, then subtract five from the total to get total-odd.
2. Add up the total score for all even-numbered questions, then subtract that total from 25 to get total-even.
3. Add total-odd and total-even, and the result is multiplied by 2.5.

**Figure 12:**
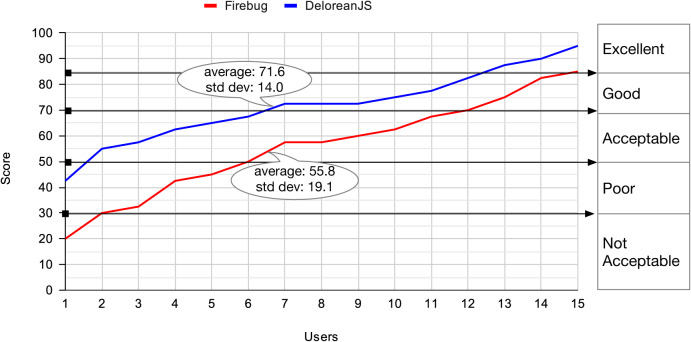
In ascending order, the SUS score for 15 participants that use DeloreanJS and Firefox’s debugger.

In an ascending order based on the score, Fig. 12 shows the usability score evaluation using SUS for each participant: 15 for each debugger. Whereas the average score for DeloreanJS is 71.6 (“Good”), the average score for Firefox’s debugger is 55.8 (“Acceptable”). Although DeloreanJS’ evaluation average is not “Excellent”, we can claim it is better than the other debugger. Additionally, we can show that three participants found the user interface of our debugger to be “Excellent”, and two participants found Firefox’s debugger to be “Not Acceptable”. Considering some of the participants’ comments (available in Spanish), we might argue that the result is because DeloreanJS’ user interface entirely focuses on JavaScript debugging. However, Firefox’s debugger integrates other aspects of a Web application, for example, the use of Cascading Style Sheets, performance, or networking.

## Conclusion

The software industry is geared toward building increasingly large and complex Web applications, implying a higher probability of introducing bugs in them. To build these applications, the JavaScript language is commonly used. To help support the implementation of applications, different debuggers are available on the Web (Barr et al., 2016; Session Stack, 2022; Raygun, 2022; TrackJS, 2022; Barton & Odvarko, 2010; JsBin, 2022; NodeJS Inspector, 2022; Azar, 2016). However, most of these offer classic breakpoint features, which can pose problems with the identification of the cause of bugs, beyond specific values leading to bugs. The use of back-in-time debuggers, like DeloreanJS, can increase the ability of developers to reason and identify the causes of bugs, for example, by navigating through timepoints and generating of multiple execution timelines, without requiring re-execution of the complete application. The functionality and usability of DeloreanJS, is presented through the use of a proof-of-concept application evaluating a MIS, and a usability evaluation of DeloreanJS’ user interface. The results of our study confirm that DeloreanJS is effective in (1) improving the understanding of bugs through scenario experimentation, and (2) providing enhanced usability by offering more information than other web debuggers currently available.

Currently, DeloreanJS faces some challenges. One of these challenges is that of *time travel paradoxes*. These paradoxes appear when a developer modifies values from a timepoint, resumes the application execution, and then navigates back to other timepoints. The problem here is that timepoints represent a snapshot of the execution within a specific context. The actions of going back-in-time and modifying values contained in a timepoint create new executions with different contexts, *i.e.,* timelines (Fig. 2). Consequently, a *future* timepoint maybe not be the result of a *past* timepoint, which may confuse a developer. To avoid these time-travel paradoxes, DeloreanJS can limit the use of future timepoints (Milner, Tofte & Macqueen, 1997) when associated with modifications at past timepoints. Another challenge is measuring *when*, in terms of programming experience, DeloreanJS (and other debuggers) can benefit developers. To address this challenge, we plan to conduct similar evaluations presented in the Usability Evaluation section with programmers with different ranges of experience in JavaScript (*e.g.,* 0–1, 2–4, and more than 4 years).

## Additional Information and Declarations

### Data Availability

The following information was supplied regarding data availability: The software is available at GitHub: https://github.com/pragmaticslaboratory/deloreanjs; Paul Leger. (2022). DeloreanJS. Zenodo. https://doi.org/10.5281/zenodo.7419790. The data (usability evaluation) is available at: https://pleger.cl/sites/deloreanjs/results.html; Paul Leger. (2022). Evaluation-of-debuggers [Data set]. Zenodo. https://doi.org/10.5281/zenodo.7402483.
